# Highly Efficient Spatial–Temporal Correlation Basis for 5G IoT Networks

**DOI:** 10.3390/s21206899

**Published:** 2021-10-18

**Authors:** Xiangping Gu, Mingxue Zhu, Liyun Zhuang

**Affiliations:** 1 Jiangsu Laboratory of Lake Environment Remote Sensing Technologies, Huaiyin Institute of Technology, Huai’an 223003, China; zlytpjs@hyit.edu.cn; 2 Faculty of Electronic Information Engineering, Huaiyin Institute of Technology, Huai’an 223003, China; zhu111903010106@126.com

**Keywords:** 5G IoT networks, compressive sensing, spatial–temporal correlation, sparse basis

## Abstract

One of the major concerns in 5G IoT networks is that most of the sensor nodes are powered through limited lifetime, which seriously affects the performance of the networks. In this article, Compressive sensing (CS) technique is used to decrease transmission cost in 5G IoT networks. Sparse basis is one of the important steps in the CS. However, most of the existing sparse basis-based method such as DCT (Discrete cosine transform) and DFT (Discrete Fourier Transform) basis do not capture data structure characteristics in the networks. They also do not take into consideration multi-resolution representations. In addition, some of sparse basis-driven methods exploit either spatial or temporal features, resulting in performance degradation of CS-based strategies. To address these challenging problems, we propose a novel spatial–temporal correlation basis algorithm (SCBA). Subsequently, an optimal basis algorithm (OBA) is provided considering greedy scoring criteria. To evaluate the efficiency of OBA, orthogonal wavelet basis algorithm (OWBA) by employing NS (Numerical Sparsity) and GI (Gini Index) sparse metrics is also presented. In addition, we discuss the complexity of the above three algorithms, and prove that OBA has low numerical rank. After experimental evaluation, we found that OBA is capable of the sparsest representing original signal compared to spatial, DCT, haar-1, haar-2, and rbio5.5. Furthermore, OBA has the low recovery error and the highest efficiency.

## 1. Introduction

Tens of billions of objects are connected to the 5G communication networks. These objects form the well-known Internet of Things (IoT), which is a promising application in future wireless networks [1,2,3]. However, 5G IoT networks face serious challenges, which are caused by the complex, variable communication environment and big data produced. Therefore, the main issue is reducing energy consumption in 5G IoT networks. Compressive sensing (CS) [4,5,6,7,8] presents some novel data-gathering strategies to reduce energy consumption in networks. According to the spatial, temporal, or spatial–temporal correlation characteristics of sensory data of 5G IoT networks, CS technique is able to recover the original senor node readings from N nodes with the help of M(M<<N) CS measurements as long as the signal can be sparsely represented in a certain transform domain [9,10]. CS is also capable of performing sensing and compression simultaneously to decrease transmission costs, aiming to save energy consumption for each node in the network.

A variety of compressive data-gathering schemes have been investigated for networks [11,12,13,14,15,16,17,18,19,20,21,22,23,24]. In reference [11], sparsity in each of the decorrelated streams is used for temporal compression. In addition, the multivariate data are characterized using multivariate normal autoregression-integrated moving-average modeling before compression. Soheil Salehi et.al. proposed an adaptive compressed sampling via multi-bit crossbar array approach to intelligently generate the CS measurement matrix using a multi-bit STO-MRAM crossbar array. In addition, energy-aware adaptive sensing for IoT was introduced. It determined the frequency of measurement matrix updates within the energy budget of an IoT device. Qiao et al. proposed a media modulation-based mMTC (massive machine-type communication) solution for increasing the throughput. This technique leveraged the sparsity of the uplink access signals of mMTC received at the base station. A CS-based massive access solution was also promoted for tackling the challenge [13]. In reference [14], novel effective deterministic clustering using the CS technique was introduced to handle the data acquisition. Han et al. in reference [15] proposed a multi-cluster cooperative CS scheme for large-scale IoT networks to observe physical quantities efficiently, which used cooperative observation and coherent transmission to realize CS measurement. However, existing sparse bases such as DCT (Discrete Cosine Transform), DFT (Discrete Fourier Transform) basis, and PCA (Principal Component Analysis) do not capture data structure characteristics in networks. As one of the statistical anomaly detection approaches, PCA can be applied to mark fraudulent transactions by evaluating applicable features to define what can be established as normal observation, and assign distance metrics to detect possible cases that serve as outliers/anomalies. However, it uses an orthogonal transformation of a set of observations of probably correlated variables into a set value of uncorrelated variables in a linear way. It serves a multivariate table as a smaller set of variables to be able to inspect trends, bounces, and outliers. In addition, the PCA method does not detect internal localized structures of original data. On the other hand, the PCA method does not provide multi-scale representation and eigenvalue analysis of data where the variables can occur in any given order. PCA achieves an optimal linear representation of the noisy data but is not necessary for noiseless observations in networks. It also does not gain multi-resolution representations. The proposed method in this paper has better performance in a noiseless environment for anomaly detection or outlier identification.

Some of the existing CS-based strategies try to exploit either spatial or temporal correlation of sensor node readings. Hence, the performance improvement brought by the CS approach is limited. Sensor node readings are generally periodically gathered for a long time. Therefore, the temporal correlation of each node can be further used. Additionally, sensor node readings have spatial correlation characteristics. Consequently, in this paper, spatial and temporal correlation features are both exploited to enhance data-gathering performance. As we know, for CS-based data-gathering methods, there are two important factors—sparse basis and measurement matrix—which should be considered. The measurement matrix includes the dense matrix [10] and the sparse matrix [24]. In reference [10], Luo et al. provided a dense matrix, which satisfied RIP. Unfortunately, this type of matrix has high computational complexity, resulting in a high cost to transform network data. Therefore, Wang et al. presented a sparse random matrix, which demonstrated that this kind of matrix had optimal K-term approximation [24]. Through many of experiments, Li et al. showed that recovery accuracy of sparse binary matrix outperformed existing sparse random matrixes [25]. As a result, the sparse binary matrix was used to gather data and reconstruct original data.

Sparse representation of sensory data aims to achieve the sparsity basis of sensor node readings. In this paper, a spatial–temporal correlation basis algorithm (SCBA) of sensory data from the detected field will be constructed in detail. Zhao et al. first adopted the transform in [26] to design a clustered compressive data aggregation scheme in networks [27]. Unlike reference [26], in this paper, according to sensory data characteristics, we design SCBA technology for 5G IoT networks. The optimal basis algorithm (OBA) is provided. At the end, we analyze the SCBA numerical sparsity using different sparsity metrics, and calculate the recovery error in view of different amounts of measurement combined with a sparse binary matrix.

The main contributions of this paper are as follows.

- We analyze various real datasets of 5G IoT networks in terms of the exponential model and rational quadratic model, respectively. It shows that sensory data have high spatial–temporal correlation features.
- In this paper, the SCBA method is put forward. In this algorithm, numerical sparsity is introduced to evaluate the performance of various sparse bases. In addition, algorithm complexity is also calculated. On the other hand, the OBA algorithm considering greedy scoring is presented. To compare the performance of the proposed SCBA with wavelet bases, the orthogonal wavelet basis algorithm (OWBA) is also presented.
- We implement a variety of experiments based on real datasets of 5G IoT networks, including noiseless and noise environments. We compare our proposed SCBA with other sparse bases in view of different numerical sparsity and various recovery algorithms. Experiments demonstrate that the novel SCBA has better performance.

The rest of the paper is organized as follows. Section 2 presents related work. Section 3 provides CS backgrounds, the network model, and two different sparsity metrics. The spatial–temporal correlation properties of sensory data are analyzed though the power exponential (PE) model and the rational quadratic (RQ) model of networks, SCBA is constructed, and OBA is proposed in Section 4. Section 5 calculates the time complex of these proposed algorithms. In Section 6, to verify the effectiveness of our presented algorithm, experiments on real datasets are carried out and related discussions are investigated. Conclusions and future work are given in Section 7. A notation table is given in the Table 1.

## 2. Related Work

Previous work related to sparse bases in networks can be sorted into the following four categories. The first is that they neither consider the spatial correlation nor consider the temporal correlation of sensory data in WSNs. For instance, DCT sparse basis [19] was used and cost-aware stochastic compressive data-gathering was proposed. A Markov chain-based model was required to characterize the stochastic data-collection process. Sun et al. [6] modeled the data loss induced by packet collisions and confirmed the corresponding compressive sensing projection matrix using the data loss pattern. Random sampling at each node was adopted and the optimal sensing probability was obtained. In the work in [6], a DFT sparse basis was used to recovery original data. Ebrahimi et al. investigated the use of unmanned aerial vehicles (UAVs) for gathering data in networks [22]. Projection-based compressive data-gathering (CDG) was attempted to aggregate sensory data. Projected nodes were chosen as cluster head nodes (CHs), while the UAV transferred that collected sensory data from the CHs to a distant sink node.

Another method is to only take into account the spatial correlation of sensory data. For example, Wu et al. [28] proposed covariance-based sparse basis. The covariance matrix was defined as follows:

(1) $$\Sigma =E(XX^{T})$$

where Σ is a real symmetric matrix, and can be represented as

(2) $$\Sigma =U\Lambda U^{Τ}$$

In reference [28], U is used as a sparse basis.

A third is to only take into consideration the temporal correlation of sensory data. Wu et al. [29] observed that the soil moisture process was relatively smooth and changed slowly, except at the onset of a rainfall. This technique tried to consider the difference between two adjacent sensory data samples, and the signal might be sparse represented. Therefore, the difference matrix was defined using Equation (3).

The fourth is to not only consider spatial correlation but also consider the temporal correlation of sensory data. Chen et al. provided a Fréchet mean estimate sparse basis [30]. In this work, both the intra-sensor and inter-sensor correlation were exploited to decrease the number of samples required for recovering of the original sensory data. It depicts that spatial and temporal correlation of a signal are considered simultaneously. Moreover, a Fréchet mean enhanced the greedy algorithm, called precognition matching pursuit (PMP). Quer et al. [31] investigated the problem of compressing a large and distributed signal of networks and reconstructed it though a small number of samples. Bayesian analysis was proposed to approximate the statistical distribution of the principal components, and to demonstrate that the Laplacian distribution provided a precise representation of the statistics of original sensory data. Principal Component Analysis (PCA) was exploited to capture not only the spatial but also the temporal correlation features of real data. In reference [32], covariogram-based compressive sensing (CBCS) was presented. In particular, Kronecker CS framework was employed to leverage the spatial–temporal correlation characteristics. CBCS performance showed that it was superior to DFT, distributed source coding, etc. It was also able to adapt efficiently and promptly to change for the signal.

(3) $$\Psi = [\begin{array}{l} -1\;\;1\;\;0\;\;\cdots \;\;0\;\;0\\ 0\;\;-1\;\;1\;\;\cdots \;\;0\;\;0\\ 0\;\;0\;\;-1\;\;\cdots \;\;0\;\;0\\ \vdots \;\;\cdots \;\;\cdots \;\;\cdots \;\;\vdots \;\;\vdots \\ 0\;\;0\;\;\;0\;\;\cdots \;-1\;\;1\\ 0\;\;0\;\;\;0\;\;\cdots \;\;0\;-\gamma \prime \end{array}]$$

Motivated by the fourth type of sparse representation basis, this paper produces SCBA aiming for the sparest representation of the sensory data in 5G IoT networks such that there is a reduction in energy consumption.

## 3. Problem Formulation

### 3.1. Compressive Sensing Overview

Compressive sensing provides a novel paradigm for signal sampling and compression in 5G IoT networks. The theory states that a sparse or compressible signal can be recovered with high accuracy from a small part of measurements, which is far smaller than the length of the original data. For instance, given an N-dimension signal vector, $X={\left(x_{1},x_{2},\ldots ,x_{n}\right)}^{T}$ describes the sensor node readings in networks with N nodes. We know that X is a K-sparse signal if there are only K(K<<N) non-zero components, or (N−K) smallest components can be ignored in X. Then, X can be expressed as follows:

(4) $$X=\Psi S=\sum_{i=1}^{N}\psi_{i}s_{i}$$

where $\Psi =[\psi_{1},\psi_{2},\ldots ,\psi_{N}]\in {ℜ}^{N}$ is given a sparse basis matrix and $S\in {ℜ}^{N}$ is the corresponding coefficient vector.

To decrease the dimensionality of X, a measurement matrix $\Phi \in {ℜ}^{M\times N}$ is adopted to achieve an M-dimensional signal $Y\in {ℜ}^{M}$, and K<M<N. In addition, the CS technique asserts that a K-sparse signal X can be reconstructed with high accuracy from M=O(Klog(N/K)) linear combinations of measurement Y. The measurement matrix can be a Gaussian or Bernoulli matrix that follows the restricted isometry property (RIP) [33].

**Definition** **1.** *(RIP [34]): A matrix Φ* *satisfies the restricted isometric property of order K* *if there exists a parameter $\delta_{K}\in (0,1)$* *so that*

(5) $$(1-\delta_{K}){\left\|X\right\|}_{2}^{2}\leq {\left\|\Phi X\right\|}_{2}^{2}\leq (1+\delta_{K}){\left\|X\right\|}_{2}^{2}$$

for all K-sparse vectors.

Candès et al. have demonstrated that reconstructing the signal X from Y can be obtained by solving an $l_{1}$-minimization problem [34], i.e.,

(6) $$\underset{X\in {ℜ}^{N}}{\min}{\left\|X\right\|}_{l_{1}}\;s.t.\;Y=\Phi X$$

Furthermore, there is a large number of recovery algorithms, including Basis Pursuit (BP) algorithm [33], (Basis Pursuit De-Noising) BPDN [33], Orthogonal Matching Pursuit (OMP) [35], Subspace Pursuit (SP) [36], Compressive Sampling Matching Pursuit (CoSaMP) [37], StagewiseWeak Orthogonal Matching Pursuit (SWOMP) [38], Stagewise Orthogonal Matching Pursuit (StOMP) [39], and Generalized Orthogonal Matching Pursuit (GOMP) [40].

### 3.2. Network Model

**Figure 1:**
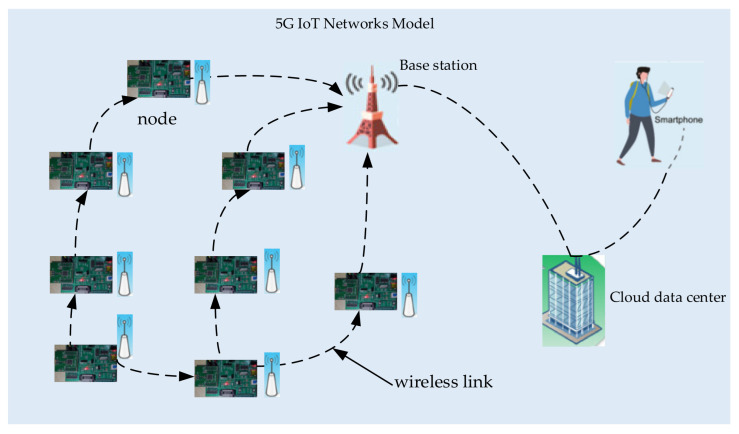
5G IoT networks model.

We consider that one multi-hop IoT network consists of N sensor nodes and one static sink node. We assume that the sensor nodes are deployed uniformly and randomly in a unit square area to periodically sample sensory data from the detected environment. The system model is described by an undirected graph G(V,E), where the vertex set V is the sensor nodes of 5G IoT networks, and the edge set E denotes the wireless links among those various sensor nodes. In addition, sensor node readings are obtained from all the nodes and transmitted to the static sink periodically. We assume that vector $X(k)={\left[x_{1k},x_{2k},\ldots ,x_{Nk}\right]}^{T}$ denotes the node readings at sampling instant k, where $x_{ik}$ represents node i’s readings. Figure 1 is the 5G IoT network model. Nodes in IoT networks transmit data by multi-hop wireless link to the base station. Finally, data are sent to the cloud data center to be processed.

### 3.3. Sparse Metrics

It is well known that sparsity K of sensor node readings X in orthogonal basis Ψ is generally measured by $l_{0}$ norm, i.e., $K={\left\|S\right\|}_{0}\;s.t.\;X=\Psi S$. In fact, there is only a small fraction of larger coefficients including most of the energy. In this section, Gini index (GI) [41,42] and numerical sparsity [43] are introduced.

**Definition** **2.** *Gini Index (GI): If the coefficient vector of signal*X*in orthogonal basis* Ψ *is* $S={\left[s_{1},s_{2},\ldots ,s_{N}\right]}^{T}$ *, which are arranged ascending order, i.e.,* $\left|s_{1\prime }\right|\leq \left|s_{2\prime }\right|\leq \ldots \leq \left|s_{N\prime }\right|$ *, where* 1′,2′,…,N′ *represent the novel indexes after reordering. Subsequently, GI is denoted as follows:*

(7) $$GI=1-2\sum_{i=1}^{N}\frac{\left|s_{i}\right|}{{\left\|S\right\|}_{1}}(\frac{N-i+1/2}{N})$$

GI implies the relative distribution of energy among the different coefficients. As can be seen from Equation (7), the value of GI is normalized and ranges from 0 and 1. It turns out that when GI is large, then the sensor node readings have only a few values that are dominated. In addition, when GI is small, readings have very few dominated coefficients. However, since $l_{0}$-norm is instability in application, alternatively, numerical sparsity is put forward. Its definition is as follows.

**Definition** **3.** *Numerical Sparsity (NS) [43]: If the coefficient vector of signal X* *in orthogonal basis Ψ* *is $S\in {ℜ}^{N\times 1}$* *, numerical sparsity (NS) of vector X* *is described.*

(8) $$NS=\frac{{\left\|S\right\|}_{1}^{2}}{{\left\|S\right\|}_{2}^{2}}$$

The ratio between ${\left\|S\right\|}_{1}^{2}$ and ${\left\|S\right\|}_{2}^{2}$ is applied to represent $l_{0}$-norm. For any non-zero coefficient vector S, $l_{1}$-norm and $l_{2}$-norm satisfy the following inequality

(9) $${\left\|S\right\|}_{2}\leq {\left\|S\right\|}_{1}\leq \sqrt{N}{\left\|S\right\|}_{2}$$

Additionally, the value of NS ranges from 1 and N, and it also has an upper bound, namely $NS\leq {\left\|S\right\|}_{0}$.

### 3.4. Spatial–Temporal Correlation Features Analysis of a Real Dataset

**Figure 2:**
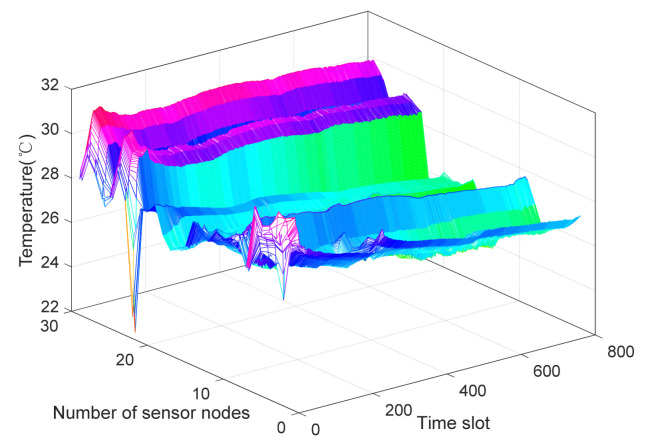
Spatial–temporal correlation features of DEI-Campaign A.

The spatial–temporal correlation properties of the various sensor nodes can be generally exploited to considerably save energy consumption in networks [44]. In this section, we extract one temperature dataset from Campaign A of DEI [45] that is representative of other datasets to approximately estimate a spatial–temporal correlation characteristic. A testbed of DEI at the University of Padova collects sensory data from 68 TmoteSky wireless sensor nodes. The sensor node hardware properties are an IEEE 802.15.4 Chipcon wireless transceiver working at 2.4 GHz, and the maximum data rate is 250 kbps. In addition, in DEI-Campaign A dataset, there are 29 nodes in total, and the frame length of sensor node readings is 781. Figure 2 plots the temperature signal features of DEI-Campaign A. The x-axis describes the time slot (frame length), the y-axis is the number of sensor nodes, and the z-axis is the corresponding temperature values of various sensor nodes. From Figure 1, we can see that most sensor node readings have a bit of variance, which are within the scope 28 °C and 31 °C. There is only a small fraction of readings with a lower value of about 22 °C. In other words, at the same sampling instant, collected data of the adjacent nodes has a high spatial correlation characteristic. When sensor nodes with high density are deployed in the detected field, as shown in Figure 2, a 3D graph has many planes. Therefore, intuitively, we consider that the real sensor datasets have a high spatial–temporal correlation.

**Figure 3:**
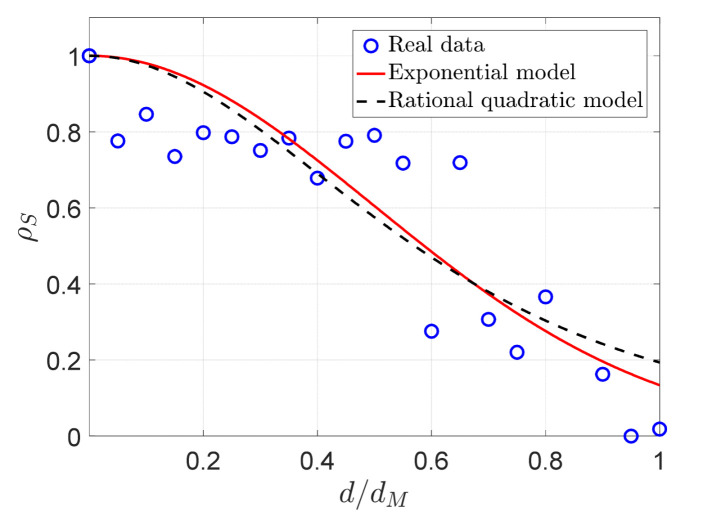
The comparison between the exponential model and the rational quadratic model.

On the other hand, we also analyze the spatial–temporal correlation features in view of theory in detail. To investigate the spatial and temporal correlation properties of the real sensor node readings respectively, we follow a similar method to that provided by Zordan et al. in reference [46]. To calculate the spatial correlation feature, we chose 29 × 781 pairs from the whole data. For each pair, we estimated its Euclidean distance d and its own spatial correlation function $\rho_{s}$ with the help of Equation (10) of reference [46]. Subsequently, we used the same approach as in [41], with 20 intervals divided for the maximum distance $d_{\max}$. Afterwards, the average spatial correlation coefficients for all pairs are calculated. Then, the relationship between spatial correlation and distance is also evaluated by the power exponential (PE) model and the rational quadratic (RQ) model. Figure 3 depicts the relationship between spatial correlation $\rho_{s}$ and the normalized distance $d/d_{\max}\in [0,1]$ of the real sensor node readings from DEI, where for the PE model, the parameters ς=0.693, and ν=1.952, while for the RQ model, ς=1.609, and ν=2. As can be seen from Figure 3, the spatial correlation of the real dataset adopted in this paper fits the PE model. Moreover, $\rho_{s}$ values of most of the blue circles in Figure 2 are larger than 0.65 or so, which indicates that it has a high spatial correlation. Nevertheless, the temporal correlation coefficients of sensory dataset are also calculated using Equation (11) in reference [46]. It turns out that the average temporal correlation coefficient of temperature of DEI-Campaign A is 0.9995, which implies that it also has a strong temporal correlation.

(10) $$\rho_{s}(p_{1},p_{2})=\frac{\mathrm{cov}(z(p_{1},t),z(p_{2},t))}{\sigma_{z}(p_{1},t)\sigma_{z}(p_{2},t)}$$

where cov(.) is the covariance function, and $\rho_{s}(p_{1},p_{2})$ is the spatial correlation function between any two points $p_{1},p_{2}$,$p_{1},p_{2}\in D$,t∈T. T is the time domain. D is the space domain.

(11) $$\rho_{T}(t_{1},t_{2})=\frac{\mathrm{cov}(z(p,t_{1}),z(p,t_{2}))}{\sigma_{z}(p,t_{1})\sigma_{z}(p,t_{2})}$$

where $\rho_{T}(t_{1},t_{2})$ is the time correlation function of any two time samples $t_{1},t_{2}\in T$.

## 4. Algorithm Details

Sparsest bases play an important role in the compressive data-gathering technique of networks. DCT, wavelet basis, and the PCA algorithm are widely used in conventional compressive data-gathering schemes. Unfortunately, these existing sparse bases do not capture intrinsic features of a signal. Take PCA, for example. PCA can obtain a global representation, where each basis vector is a linear combination of all the original data. It is not easy to detect internal localized structures of original data. On the other hand, the PCA method does not provide multi-scale representation and eigenvalue analysis of data where variables can occur in any given order. In addition, PCA achieves an optimal linear representation of noisy data but is not necessary for noiseless observations in networks. Therefore, when the number of observations is far greater than the number of variables, the principal elements may be interfered with by the noise. IoT networks fall into this category. In other words, the number of sensor node observations is no less than the amount of sensor nodes in the networks. Thus, in this paper, motivated by hierarchical clustering tree and wavelets [25], a novel algorithm that not only captures localized data structure characteristics, but also gains multi-resolution representations, is presented. SCBA is summarized in Algorithm 1.

In Algorithm 1, there are three stages that include the calculation of the two most similar sum variables, building a hierarchical tree of 2 × 2 Jacobi rotations and constructing a basis for the Jacobi tree Algorithms.

**Stage1:** For this algorithm, in step 1, covariance matrix $\Sigma_{ij}$ is the general covariance, which is shown in Equation (12). The correlation coefficients $\rho_{ij}^{}$ is described using Equation (13), and the similarity matrix is represented as Equation (14).

(12) $$\Sigma_{ij}=E[(x_{i}-E(x_{i}))(x_{j}-E(x_{j}))]$$

(13) $$\rho_{ij}^{}=\frac{\Sigma_{ij}}{\sqrt{\Sigma_{ii}\Sigma_{jj}}}$$

(14) $$SM_{ij}=\left|\rho_{ij}\right|+\gamma \left|\Sigma_{ij}\right|$$

where γ≥0. Subsequently, in step 2, we calculate the most similar sum variables based on the similarity matrix $SM_{ij}$. However, at the initial stage 1, when input dataset is X, for instance, the size of an extracted matrix from the temperature of the DEI-Campaign A is 29∗781. If we calculate correlation coefficients between different rows for each column vector, it means that the spatial correlation is considered. When we calculate correlation coefficients between different columns for each row vector, it shows that the temporal correlation is also taken into account. In application, for a detected environment of 5G IoT networks, we choose datasets as input variables X of several minutes frame length which are enough to explore the intrinsic features of sensor node readings. By means of these collected data, we can design a SCBA schedule. Consequently, in the following compressive data-gathering scheme, we can combine the measurement matrix with the given reconstruction algorithm to recover the original signals in the sink node of networks.

**Stage2:** Steps 3–24 mainly construct a tree of Jacobi rotations. In step 4, variable T is applied to store Jacobi rotations matrix, while theta denotes rotation angle. Variable PCindex is the order of the principle component. Next, Step 7 initializes the related parameters of the algorithm. For the loop, steps 8–24 calculate Jacobi rotations for each level of the tree. Variable CM and cc represent covariance matrix $\Sigma_{ij}$ and the correlation coefficient matrix $\rho_{ij}^{}$, respectively. By naming the newJacobi function, we accomplish a change of basis and new coordinates, which corresponds to steps 9–15. Steps 16–23 reveal various approaches of variable storage. Step 16 is the number of new variables for sum and difference components.p1 and p2 represent the position of the 1st and the 2nd principal components at step 17, respectively. So far, it has constructed a Jacobi tree.

**Stage3:** Then, in the following steps, we will produce the orthogonal basis for the aforementioned Jacobi tree algorithm. The loop of 26–34 is the core of the orthogonal basis algorithm, which repeats until lev achieves the maximum maxlev. However, R denotes a 2 × 2 rotation matrix. The two principal components yy(1) and yy(2) are stored in variables sums and difs, respectively, that correspond to lines 29–33. It is worth stressing that sums is the fraction of basis functions of subspaces $V_{1},V_{2},\ldots ,V_{m-1}$, and difs is the basis functions of subspaces $W_{1},W_{2},\ldots ,W_{m-1}$. In addition, the spatial–temporal correlation basis algorithm is similar to standard multi-resolution analysis: The SCBA algorithm provides a set of “scale functions”. Those functions are defined on subspaces $V_{0}⊃V_{1}⊃\ldots ⊃V_{L}$ and a group of orthogonal functions are defined on residual subspaces ${\left\{W_{l_{k}}\right\}}_{l_{k}=1}^{L}$, where $V_{l_{k}}⊕W_{l_{k}}=V_{l}{}_{{}_{k}-1}$ such that they achieve a multi-resolution transformation. Thus, the orthogonal basis is the concatenation of sums and difs (lines 35–39).

**Figure 4:**
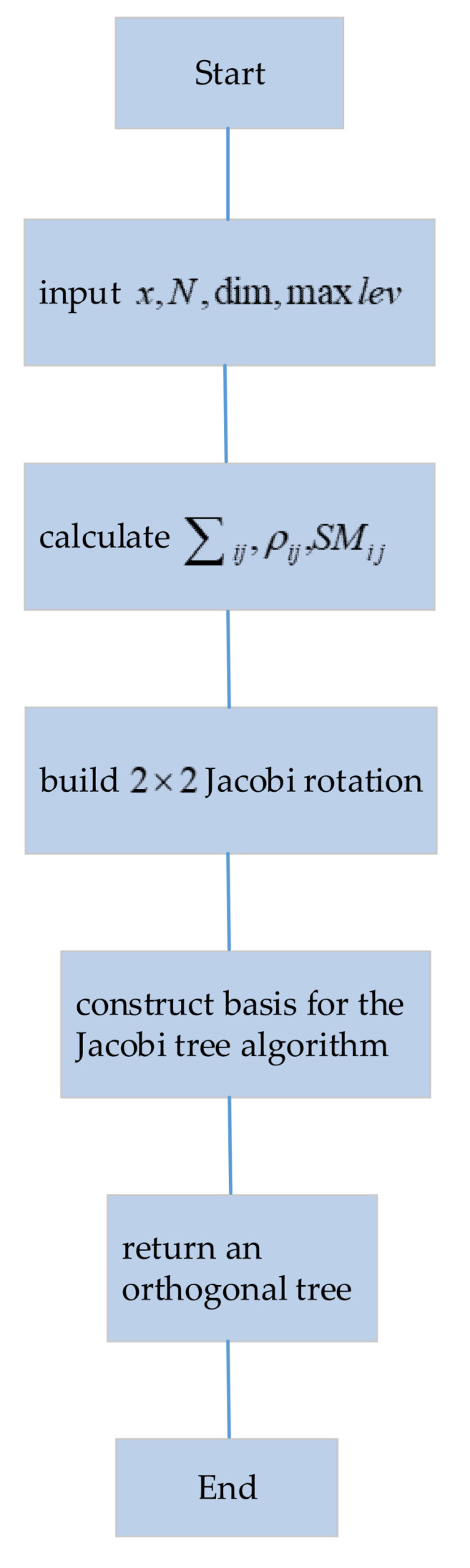
The flow chart of SCBA.

However, in Algorithm 1, the default basis selection is the maximum-height tree. The choice results in a fully parameter-free decomposition of the original data. In addition, it is also specifically for the idea of a multi-scale analysis. In practice, for a compressive data-gathering technique for 5G IoT networks, we alternatively select any of the orthogonal bases at various levels of the tree. The algorithm provides an approach that is inspired by the idea in reference [45]. We assume that the original data $x_{i}\in {ℜ}^{q}$ is a q-dimensional random vector. We suppose that the candidate orthogonal bases are $Basis_{0},Basis_{1},\ldots ,Basis_{p-1}$, where $Basis_{l_{k}}$ denotes the basis at level $l_{k}$ of the tree. Subsequently, we find the best sparse representation for the original signal. Here, in Algorithm 2, scoring criteria are applied to measure the percentage of explained variance for the selected coordinates. Consequently, greedy scoring and choice method is presented in the following Equation (15).

(15) $$score(W_{i})=\frac{E\{\left|W_{i}\right|\cdot X\}}{E\{{\left\|X\right\|}^{2}\}}$$

where for an orthogonal basis $Basis=(W_{1},W_{2},\ldots ,W_{p})$, each vector $W_{i}$ is assigned an energy score based on the above Equation (15). Therefore, the optimal basis is the basis with the highest energy score. In Algorithm 2, line 3 describes the value of the molecule, and line 5 represents the value of the denominator of $score(W_{i})$. Of course, in Algorithm 2, the other two sparsity measurement strategies are taken to evaluate the performance of the spatial–temporal correlation sparse basis. Line 6 and line 7 are 1-norm and 2-norm, respectively. They are used to compute GI and NS, respectively, and steps 10–11 of Algorithm 2 are the GI index and NS evaluation approaches. Then, line 12 arranges the energy score in Equation (15) in descending order such that we find the best orthogonal basis with the maximum energy score. At the end, lines 13–16 obtain the optimal basis. In addition, the flow chart of SCBA is shown in Figure 4. The main steps of SCBA input the needed parameters, calculating the two most similar sum variables, building a hierarchical tree of 2 by 2 Jacobi rotations and constructing a basis for the Jacobi tree algorithm.
**Algorithm 1** The spatial–temporal correlation basis algorithm with highly efficient (SCBA)**Input:**X, dim, N (total number of observations), maxLev, $l_{k}$**Output:** return an orthogonal basis% *calculate the two most similar sum variables*1: calculate covariance matrix $\sum_{i}{}_{j}$, correlation coefficients $\rho_{ij}$, similarity matrix $SM_{ij}$2: obtain the two most similar sum variables based on $SM_{ij}$% *build a hierarchical tree of 2 by 2 Jacobi rotations*
3: Z←zeros(J,3)4: T←cell(J,1)5: theta←zeros(J,1)
6: PCindex←unit8(zeros(J,2))7: initialization8: *for*
lev←1 to J9: $\left[CM_{new},cc_{new},\max cc,componet]\leftarrow newJacobi(CM,cc,\right)$10:   dist←(1−maxcc)/2
11:   Z(lev,:)← [double(nodes(component)),dist]12:   T{lev}←R13:   theta←th14:   PCindex←unit8(idx)15:   CM←CMnew,cc←ccnew16:   pind←componet(idx)17:   p1←pind(1),p2←pind(2)18:   va(pind)←unit16( [dim+lev,0])19.   dlables(p2)←unit16(lev)20.  maskno← [maskno,p2]21:   PC_ra(lev)←CM(p2,p2)/C(p1,p1)22:   Zpos(lev)←unit16(component)23:   ad(lev,:)←dlables′,ad(lev,:)←va24: ***end***% *construct basis for the Jacobi tree algorithm*25:   sums←zeros(maxlev,m),difs←zeros(maxlev,m)26: ***for***
lev←1 to maxlev27:   s1←tfilt(Zpos(lev))28:   R←T{lev}29:   yy←R′×s130:   filt(Zpos)←yy31:   yy←yy(PCindex(lev,:),:)32:   sums←yy(1,:)33:   difs←yy(2,:)34: ***end***35: ***if*** nargin < 436: basis← [sums(J,:);filpud(difs(J)]37: ***else***38: basis← [tmp(va,:);flipud(difs)]39: end

**Algorithm 2** optimal basis algorithm with greedy scoring (OBA)**Input:**X, basis**Output:** the best Treelet orthogonal basis: BestTreelet1: calculate coeff12: energy←coeff1.*coeff13: ave←mean(energy)4:***if***
nargin<45: av_norm←mean(sum(X.*X,2))6: av_norm1←(1−norm).^27: av_norm2←(2−noram).^28: ***end***9: ave1←ave/av_norm10: calculate GI index using Equation (4)11: calculate NS by using Equation (5)12:  [ave1,order]←sort(ave1)13: *if*
nargout>214: score←sum(ave1(1,k1))15: *end*16: BestTreelet←basis(order,:)

To demonstrate the efficiency of SCBA, in Section 6, we perform plenty of comparison experiments including spatial, DCT, haar-1, haar-2, and rbio5.5 bases. However, since the standard wavelet algorithm is not an orthogonal basis, Algorithm 3 proposes the OWBA scheme with a similar idea in reference [47]. In Algorithm 3, step 1 takes the rbio5.5 algorithm, for example, by means of filtering, and decomposes out the high and low filter coefficients. Line 2 calculates the length of the filter, and line 3 and line 4 obtain the maximum and minimum of the observation vectors, respectively. Step 5 is the initialization of the wavelet orthogonal basis. The loop of steps 6–18 aims to construct the orthogonal matrix. It is noted that the length of the signal is the integer power of 2 that is shown in step 7. Hence, in the subsequent experiment, the frame lengths of data on rbio5.5 and haar are chosen as the integer power of 2. Lines 8–9 construct two vectors. Nevertheless, in the coming loop, the aforementioned vector in lines 8–9 is circle-shifted (step 10–13). Finally, we generate the orthogonal matrix, namely the wavelet orthogonal basis wob (lines 14–17). As a result, OWBA returns an orthogonal basis until the variable i achieves the maximum, i.e., rmax.
**Algorithm 3** orthogonal wavelet basis algorithm (OWBA)**Input:** original data X, measurement size M, FLen(frame length of data), sparsity K**Output:** wavelet orthogonal basis: wob1.   [h,g]←wfilters(′rbio5.5′)2.  Length←length(h)3.  rmax←log2(FLen)4.  rmin←log2(FLen)+15.  wob←16. ***for*** i←rmin to rmax7.  nn←2^i8.  p1←sparse( [h,zeros(1,nn−FLen)])9.  p2←sparse( [g,zeros(1,nn−FLen)])10.   ***for***
j←1 to nn/211.    p1←circshift(p1′,2*(j−1))′12.    p2←circshift(p2′,2*(j−1))′13.   ***end***14.  w1← [p1;p2]15.  mm←2^rmax−length(w1)16.  w←sparse(w1)17.  wob←wob*w18. ***end***

## 5. Theoretical Analysis

### 5.1. Time Complexity of Algorithm

In this section, we analyze the complexity of the proposed three algorithms on a usual dataset with N sensor nodes (observations) and FLen frame length (variables). In Algorithm 1, stage 1 is an exhaustive search for the most similar sum variables [26]; in fact, step 2 of SCBA is the optimal processing stage. Hence, the overall complexity is $ct+O(L\times FLen^{2})$ operations, where ct parameter is the cost of calculating the covariance matrix $\Sigma_{ij}$ by using the singular value decomposition, i.e., $ct=O(\min(N\times FLen^{2},FLen\times N^{2}))$, and L is the height of the tree. Additionally, stage 2 mainly performs a local change and stage 3′s task is storing the 1st principal component and 2nd principal component. As a result, the complexity of the algorithm can be decreased to ct+O(FLen×N). It is noted that the complexity of the algorithm depends on the data size. As the size of the data increases, the complexity of the algorithm increases. Therefore, it is very important to select probable data size to design the algorithm.

For OBA algorithm, steps 1–3 calculate the energy of observations, so the time complexity is O(N×FLen). Steps 5–7 obtain the average value, 1-norm and 2-norm, the corresponding time complexity is O(FLen×N). The time complexity of implementation GI index of step 10 is also O(FLen×N). However, the complexity of NS sparsity measurement of step 11 is $O(FLen^{2})$. For the residual steps, the complexity is O(FLen×N). Thus, the overall complexity is $O(\min(FLen\times N,FLen^{2}))$.

For the OWBA algorithm, in terms of the loop of steps 6–18 (not including inner loop: steps 10–13), the time complexity is O(logFLen). For steps 10–13, in the worst case, the time complexity is $O((2^{\log FLen})/2)=O(FLen)$. Then, the overall time complexity of steps 6–18 (extra loop and inner loop) is O(FLen)×O(logFLen)=O(FLenlogFLen).

### 5.2. The Proposed SCBA Has Low Numerical Rank

**Theorem** **1.** *If similar matrix*SM*is constructed using Equation (12) and we build a hierarchical tree of* 2×2 *Jacobi rotations, then the sparse operator has low numerical rank.*

**Proof:**  We mainly prove the basis generated in Algorithm 1 can make our real sensor data sparse in this section. First, the eigenvalues of general covariance matrix $\Sigma_{ij}$ is analyzed. In the presented Algorithm 1, we take the temperature of DEI-Campaign A; for example,781 frame lengths of sensor data are chosen to calculate the covariance matrix. We assume that SCBA basis $\Psi_{T}=[\psi_{1},\psi_{2},\ldots ,\psi_{Flen}]$ and $\Lambda =diag\{\lambda_{1},\lambda_{2},\ldots ,\lambda_{FLen}\}$ are a real symmetric matrix. According to Equation (12) and Equation (13), we can conclude that the correlation coefficient matrix is also a real symmetric matrix. Then, similarly, based on Equation (14), a similarity matrix is also a real symmetric matrix. Subsequently, when we find the most similar sum variables, we implement a local PCA on this pair of variables such that a Jacobi rotation matrix can be calculated. The transformation corresponds to a change of new coordinates $x^{\left(l\right)}=J^{T}x^{\left(l-1\right)}$, where J is Jacobi rotation matrix. In other words, $\Sigma^{\left(l\right)}=J^{T}\Sigma^{\left(l-1\right)}J$. For a real symmetric matrix, singular values are absolute values of its corresponding eigenvalues, and the singular values ranges from 0 to 1. With the increase of decomposition level, singular values gradually become small. Based on the definition of numerical rank in reference [48], we point out that Treelets operate a numerical rank with parameters $\left(\xi_{1},\xi_{2},\varepsilon \right)$ if and only if $\sigma_{r}\geq \xi_{1}>\varepsilon \geq \sigma_{r+1}$. Thus, when the two $\xi_{1}$ and $\xi_{2}$ are fixed, the value of numerical rank reduces. Therefore, the proposed SCBA method has low rank.

## 6. Experiments Results and Discussions

### 6.1. Rank Experiment Settings

In this section, we implement the experiments based on real datasets. We choose four different scenarios that are extracted from the temperature of DEI-Campaign A [45], the temperature of OrangeLab-Campaign A [49], the soil moisture of EPFL-Campaign A [50], and the voltage of DEI-Campaign B [45]. For instance, the data of 29 nodes × 781indicates that 781 temperature sample values are captured from 29 nodes during the period 19–22, March 2009. The number 29 is the row of the data matrix, while number 781 demonstrates the column of data matrix. These projects are deployed in campus, indoor, and urban environments. The properties of these datasets are summarized in Table 2. These experiments are performed on the Matlab 2016a platform on a PC. According to the SCBA scheme in Section 4, first, we evaluate the performance of the five various spatial–temporal correlation bases. Secondly, in the light of GI and NS metric, we compare the OBA algorithm with the other five sparse basis: spatial, DCT, haar-1, haar-2, and rbio5.5 wavelet orthogonal bases. In addition, we represent sensory real data on the above five different sparse bases and the proposed OBA. On the other hand, we reconstruct the original data (aforementioned real datasets in Table 2) using the different sparse bases and recovery algorithms. In addition, we carry out numerous comparison experiments in view of reconstruction error.

### 6.2. Evaluation of SCBA

**Figure 5:**
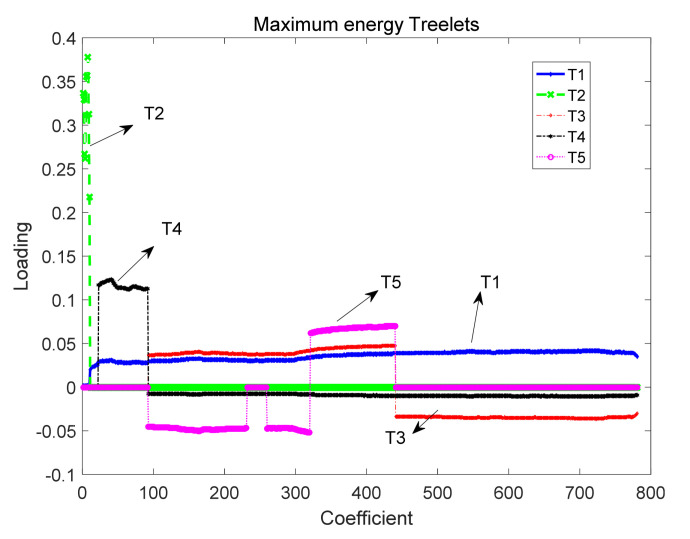
The five different SCBA bases with high energy.

We now analyze the performance of the proposed SCBA based on the first dataset. Figure 5 plots the five spatial–temporal correlation bases with the highest energy, where the *x*-axis denotes the frame length of signal, and the *y*-axis is the loading of different bases. As shown in Figure 5, $T_{1},T_{2},T_{3},T_{4},T_{5}$ are the five different bases with the energy of ascending order respectively, i.e., $T_{1}>T_{2}>T_{3}>T_{4}>T_{5}$. It is noted that the loading value is normalized and ranges from 0 to 1. Obviously, within the overall frame length, the peak of $T_{1}$ is about 0.05 or so, and the loading value of each coefficient is greater than 0. Although the maximum of $T_{2}$ is 0.38 or so, which is approximately 10 times that of $T_{1}$’ s maximum, it only concentrates on the scope of 0 to 10. When the frame length is greater than 10, the loading of $T_{2}$ is close to 0. However, during the whole frame length, for the loading of $T_{3}$, $T_{4}$ and $T_{5}$, there is a fraction of loading of coefficients less than 0. Consequently, the loadings of the three bases are no higher than $T_{1}$ or $T_{2}$.

**Figure 6:**
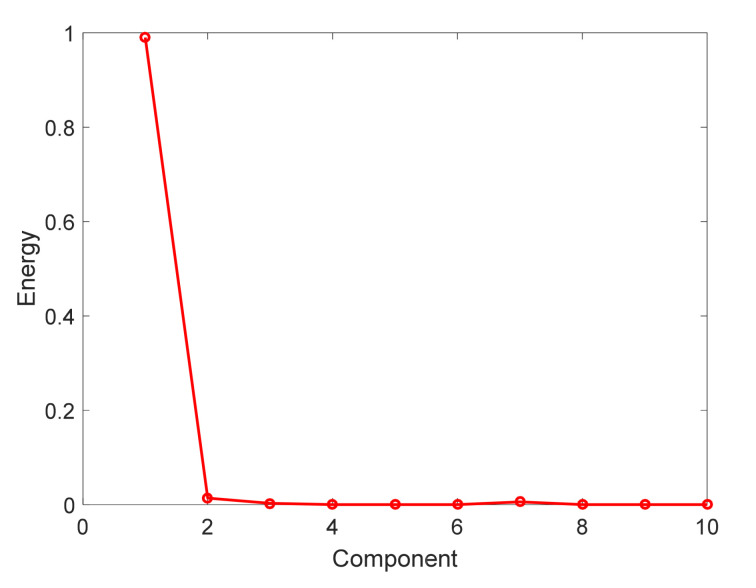
Energy distribution of principal component of the proposed SCBA.

Figure 6 plots the energy distribution of the proposed SCBA schedule. From the graph, we can see that the first component concentrates most of energy of basis which is 0.9901. In addition, the energy of the second component is about 0.0140, the residual components are close to 0. Therefore, we consider that the proposed OBA is optimal.

### 6.3. Representation of Sensory Datasets on the Various Sparse Bases

**Figure 7:**
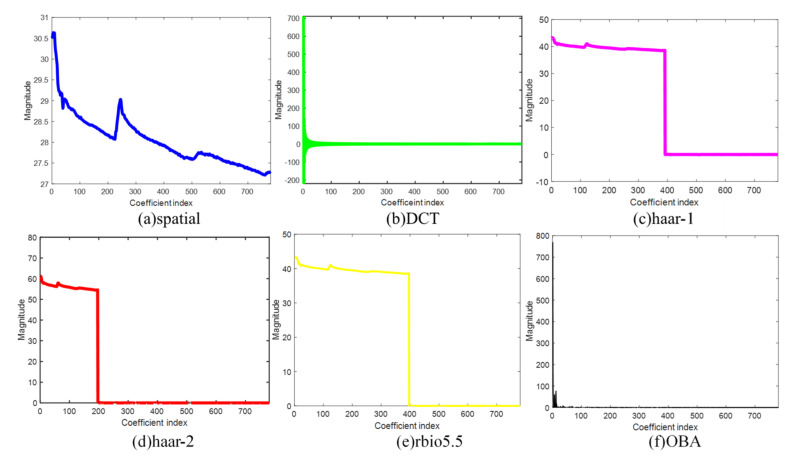
Sparsity performance of temperature of DEI-Campaign A in six various sparse bases.

**Figure 8:**
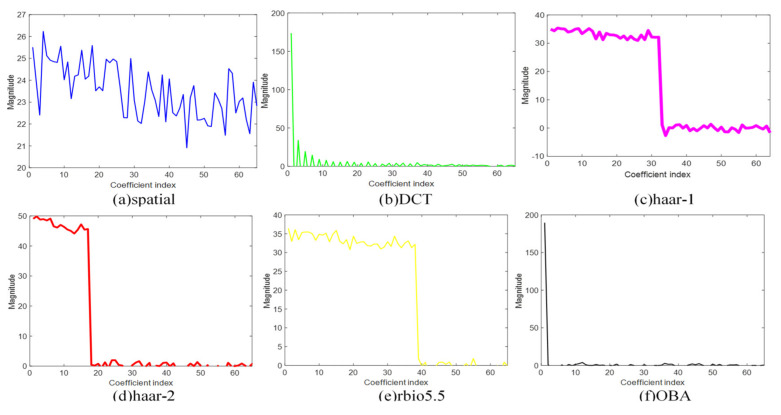
Sparsity performance of temperature of OrangeLab-Campaign A in six various sparse bases.

**Figure 9:**
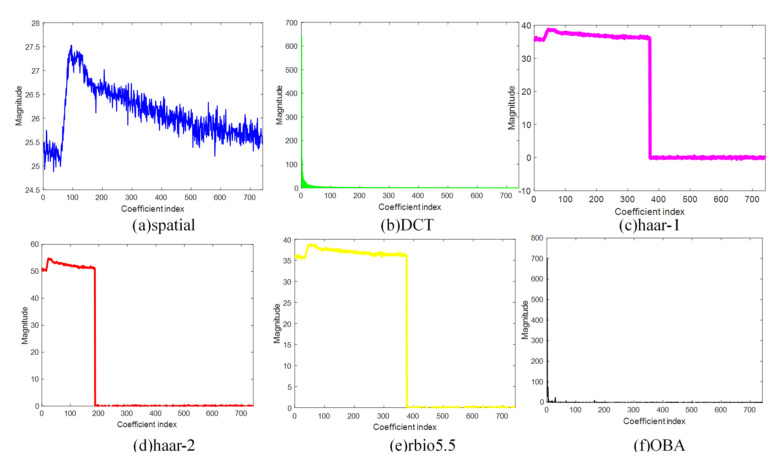
Sparsity performance of soil moisture of EPFL-Campaign A in six various sparse bases.

**Figure 10:**
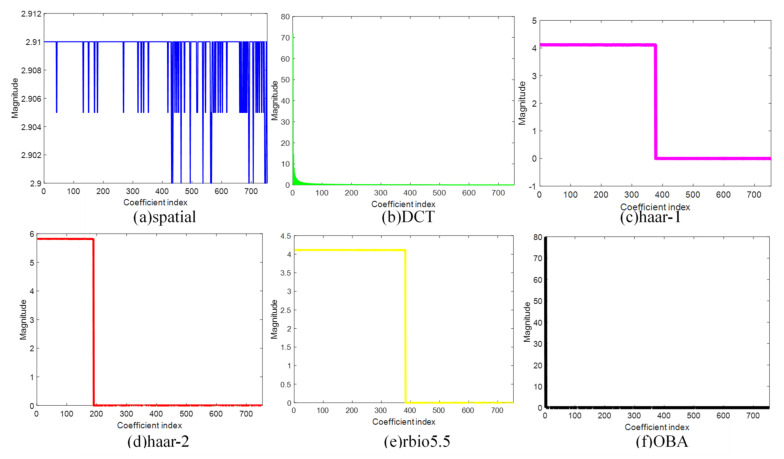
Sparsity performance of voltage of DEI-Campaign B in six various sparse bases.

In the experiment, to validate the efficiency of the proposed OBA algorithm, we compare it with the other sparse bases: spatial, DCT, haar-1, haar-2, and rbio5.5. Figure 5, Figure 6, Figure 7 and Figure 8 are the sparsity results of temperature of DEI-Campaign A, temperature of OrangeLab-Campaign A, soil moisture of EPFL-Campaign A, and voltage of DEI-Campaign B, respectively. In Figure 7, we select the first sensor node’s readings with the frame length FLen=781 to sparse represent. It is noted that haar and rbio5.5 orthogonal basis are obtained using the proposed Algorithm 3 in Section 4. As can be seen from Figure 7a, the maximum is about 30.6 of the spatial basis, and the graph resembles the original signal for the spatial basis is an identity matrix. In some senses, spatial basis is not able to sparse sensory data. For Figure 7b, the maximum is about 700, and has a small fraction of non-zero coefficients, i.e., the energy of most of coefficients is approximately zero. In contrast, the DCT basis has better sparsity performance. Similarly, haar-1, haar-2, and rbio5.5 in Figure 7 can also make the original sensor node readings sparse. However, the number of non-zero coefficients of haar-1 and haar-2 basis are far larger than DCT in Figure 7b. It is obvious that the amount of DCT non-zero coefficients can be 200 or so, and the whole length of coefficients is 781. In comparison to haar-2 basis in Figure 7d, rbio5.5 maximum is about 42, which is less than the haar-2 maximum of 60. Moreover, the number of non-zero coefficients of rbio5.5 is about twice that of haar-2′s. Hence, from Figure 7d,e, we can conclude that the former’s performance is worse than the latter. However, for OBA, its maximum is 780 or so. In addition, the number of non-zero coefficients is about 20, i.e., the energy of the residual 761 coefficients is also close to zero. From the above analysis, we draw a conclusion that the proposed basis in Figure 7f is the sparsest basis among the six various bases. From the simulation results of Figure 8, Figure 9 and Figure 10, we can see that as a whole, spatial basis does not make sensory real data sparse. The sparsity performance of DCT is superior to haar-1, haar-2, and rbio5.5 wavelet bases. Although the efficiencies of DCT are better than the wavelet basis, they are worse than the proposed OBA. In addition, the advantage of the proposed Algorithm 2 is evident, compared with the others.

### 6.4. Comparison Experiments in Terms of GI and NS Metrics

To investigate the robust performance of the proposed OBA algorithm, in this section, we perform extensive experiments in view of GI and NS metrics introduced in Section 3. These simulation results are listed in Table 3. Table 3a–d are the evaluation results of temperature of DEI-Campaign A, temperature of OrangeLab-Campaign A, soil moisture of EPFL-Campaign A, and voltage of DEI-Campaign B, respectively. As shown in Table 3a, GI of spatial, DCT, haar-1, haar-2, rbio5.5, and OBA are 0.0118, 0.2526, 0.5077, 0.7566, 0.5268, and 0.7842, respectively. Based on the analysis of the GI metric in Section 3.3, it suggests that the larger the GI value, the better the performance of the presented algorithm. Therefore, we can demonstrate that the proposed Algorithm 2 has the best performance compared to the other five sparse bases as described in Table 3a. The performance of the rbio5.5 basis is better than the haar-1. However, the GI value of haar-2 is greater than rbio5.5. In addition, the efficiency of DCT is worst among the five techniques except for spatial basis. This phenomenon is contrary to the conclusion obtained in Figure 7 in Section 6.3. It is for this reason that we take different sparsity metrics. However, when the NS metric is used, the performance of DCT is slightly worse than the best basis, which is the proposed OBA. From Table 3a, we can see that the NS metrics of them are 2.8856, 13.3167, 196.1361, 391.4284, 395.8623, and 780.6154 in ascending order. Therefore, the simulation results of the other bases—spatial, DCT, haar-1, haar-2, rbio5.5 and OBA—are in accordance with the conclusion provided in Section 6.3. It demonstrates that the value of the NS metric is inversely proportion to the efficiency of bases algorithms. In other words, the robust performances of all the sparse bases are OBA > DCT > haar-1 > haar-2 > rbio5.5 > spatial in descending order. The best value is marked in boldface in Table 3.

In Table 3b, it is obvious that the GI metric of OBA is the best of all the sparse bases at 0.7809, which is slightly better than the haar-2 of 0.7585. The efficiency of the haar-1 basis is slightly better than DCT, by contrast. Then, the performance of the rbio5.5 wavelet basis is worse than the haar-2 basis. The worst of them is the spatial basis, whose GI is 0.0292. Nevertheless, in terms of NS metric, the proposed OBA displays a superior result compared with the other five bases. The NS metric of Treelets is 1.7180, which is the smallest value of DCT at 6.8109, haar-1 at 33.3158, haar-2 at 18.5259, rbio5.5 at 39.4684 and spatial at 64.8315. This phenomenon is line with Table 3a). Similarly, it also again demonstrates that the NS value is inversely proportional to the effect of the basis. Hence, it is evident that the spatial basis has the most terrible performance, while the proposed OBA basis has the most advantage.

In view of Table 3c, the robust performance of OBA is a bit better than the haar-2 basis in terms of GI metric. In addition, the performance of haar-1 is worse than rbio5.5. However, the efficiency of wavelet bases is still worse than Treelet. DCT has a good result, whose GI is 0.2266, which is the lowest value. It is worse than the spatial value of 0.4942. It can be shown from Figure 6 that the sparsity performance of DCT is far better than the spatial basis. Thus, we consider that the GI metric does not precisely demonstrate the efficiency of the sparse basis, while in Table 3c, the value of DCT (13.3031) is inferior to the proposed Treelet (2.9541) which is identified by the simulation result in Figure 6. The case also verifies the instability of the GI metric in application.

In Table 3d, we can observe that the spatial basis has a minimum 2.5089 × 10^−4^ of GI metric and NS is 753.9996. In practice, the NS metric indicates the quantity of non-zero coefficients of the represented sensory signal. For instance, in Table 3d, NS is 753.9996, which is approximately equal to the signal length of 754. Likewise, the NS values in Table 3a–c are 780.6154, 64.8315, and 741.6826, respectively, which are close to 781, 65, and 742 of the frame length of extracted simulation datasets. In Table 3d, the GI value of the proposed OBA is 0.9820, which is approximately 1. It also declares that the novel basis is the sparsest basis among the six basis algorithms. The performance of DCT is not better than haar-1. GI of haar-2 is 0.7516, which is higher than rbio5.5 of 0.5196. Nevertheless, the value of the corresponding NS metric of the former is also less than the latter. Their results are in contrast. As a whole, different evaluation metrics achieve different results, but in this paper, we draw a conclusion that NS metrics in Table 3 have identical experiment results to Figure 5, Figure 6, Figure 7 and Figure 8, while the GI metric has a bit of deviation. In brief, the NS metric has a higher accuracy than the GI metric. It can be seen from the above figures and tables that the OBA algorithm has the most concentrated energy, the smallest NS value, and the largest GI value, i.e., when the OBA algorithm for compressed data collection and transmission is used, it will consume less energy and improve the performance of the network.

### 6.5. Reconstruction Error Results and Analysis

In this section, two various recovery algorithms are taken into consideration. The BPDN algorithm is the noise environment (σ=0.05), and the GOMP algorithm is the noiseless case. In addition, the measurement matrix is the sparse binary matrix with a fixed number of non-zero elements in each column. For the proposed Treelets sparse basis, related recovery errors using BPDN are given in Table 4, and recovery errors using the GOMP algorithm are depicted in Table 5. Recovery error is defined as follows:

(16) $$error=\frac{{\left\|X-\hat{X}\right\|}_{2}}{{\left\|X\right\|}_{2}}$$

In Table 4, in the first dataset, i.e., temperature of DEI-Campaign A, the number of non-zero elements is d=60. Similarly, in the third and fourth datasets, the measurement matrix has the same amounts of non-zero entries in each column. However, in the second dataset, d′=10. In the first dataset, the frame length is 781, K=60. Here, we assume that the relative error is less than 1, and we consider that it can recover original data. As can be seen from Table 4, for the first dataset, with the increase of measurement M, recovery error gradually decreases. In particular, when the amount of measurement is equal to 300, the relative error is 0.9592, i.e., the proposed OBA can recover the original signal. For instance, when the measurement M is 350, the error is 0.7463. However, when the measurement achieves the maximum in Table 4, the error is only 0.4385, which is less than half of 0.9592. For the second datasets, the temperature of OrangeLab-Campaign A, when the measurement is not larger than 20, it is unable to reconstruct original data. Take M=10, for example—its error is 2.7764. As the measurement increases, the recovery error of the proposed OBA along with the sparse binary measurement matrix becomes smaller and smaller. For example, when the measurements are 25, 30, 35, 40, 45, and 50, their corresponding relative errors are 0.9650, 0.8080, 0.7198, 0.6355, 0.5000, and 0.4731, respectively. Moreover, for soil moisture of the EPFL-Campaign A, the original signal cannot be reconstructed until the measurement is 60. From Table 4, when the measurement equals 50, the error is 1.0268, which is greater than 1. In contrast, 0.7936 of M=70 is far smaller than 0.9443 of M=60. In addition, for M=100, the error is only 0.4154. For the last dataset, when the measurement is the minimum, the relative error is 1.5541. It means that the novel OBA and sparse binary measurement matrix are unable to recover the original signal. As shown in Table 3, if we set measurement M at 50, the error of the proposed OBA algorithm is less than 1, i.e., 0.9252. In addition, when the measurement M=60,70,80,90,100, the errors are 0.8494, 0.7387, 0.5565, 0.5427, and 0.3943, respectively.

Table 5 depicts the relationship between reconstruction errors of the four different datasets and the measurement M using the GOMP algorithm. The parameter d′ in a sparse binary matrix, and the sparsity K and frame length of signal are the same as aforementioned Table 4. In the DEI-Campaign A, when the amount of measurement M is greater than 550, GOMP can recover the original signal. However, in terms of the BPDN algorithm, when the number of measurements M is 300, the original signal can be reconstructed. BPDN takes noise into account and therefore has better recovery performance. In the second dataset, the temperature of OrangeLab-Campaign A, when the measurement M is only about half of the frame length, GOMP can recovery the original signal with high accuracy. In comparison to BPDN, in view of the same measurement M, recovery probability of BPDN is higher than GOMP, such that when M = 35, the former is 0.7198, while the latter is 0.8384. In addition, it is noted that as the measurement M gradually increases, in terms of theory, the recovery error should steadily decrease. Nevertheless, in the GOMP algorithm, the error of the measurement M = 40 is higher than M = 35. The reason for that is that the measurement matrix uses a sparse binary matrix whose non-zero entry position is not fixed but random. For the coming third and fourth datasets, the original signal can be recovered if the measured value is equal to 80. For soil moisture of EPFL-Campaign A, when the measurement reaches the maximum, the relative error is 0.7216. In addition, for the last dataset, the smallest error is obtained when the measurement is 100. In brief, there is a big gap between BPDN and GOMP in terms of recovery accuracy. In practice applications, we should choose an appropriate reconstruction algorithm to accomplish compressive data-gathering in 5G IoT networks.

## 7. Conclusions and Future Work

In the paper, we put forward the spatial–temporal correlation SCBA algorithm and the OBA choice scheme. Theoretical analyses reveal that SCBA, OBA, and OWBA algorithms have low computation complexity. On the other hand, we also prove that the presented SCBA has low numerical rank. The experimental results show that the sensor node readings on the SCBA algorithm are sparsest in comparison to the other five sparse bases in light of the GI and NS sparsity metric. Thus, CS-based data-gathering technology using the SCBA algorithm will transmit data with less energy consumption. It will also affect the performance of 5G IoT networks. Nevertheless, in the noise environment, the BPDN algorithm is applied to reconstruct the original signals. Comparatively speaking, we observed that the proposed approach has robust performance. On the other hand, in the noiseless case, the GOMP algorithm is used, where similar experimental phenomena are discovered because of the novel algorithm taking advantage of the intrinsic distinction of the sensory data in 5G IoT networks.

This paper only discusses and analyzes how to generate the sparse basis, and is unable to study the construction of the measurement matrix. In future work, an energy-efficient data-gathering scheme with combination of the sparsest basis with an optimal measurement matrix should be designed to enhance the performance of 5G IoT networks. On the other hand, in future work we are planning to implement the approach in hardware platforms and considering the mobility of sensor nodes in 5G IoT networks.

## Figures and Tables

**Table 1:** Notation descriptions.

Name	Notation
M	CS measurements
N	the number of nodes
X	N-dimension signal vector
K	the number of sparse signals
Ψ	sparse basis matrix
Φ	measurement matrix
S	coefficient vector
G(V,E)	an undirected graph
V	vertex set
E	wireless link
ρ	correlation function
Σ	covariance matrix
${\left\|\right\|}_{1}$	1-norm
${\left\|\right\|}_{2}$	2-norm

**Table 2:** Details of Datasets in 5G IoT Networks.

Name	Time Period	Physical Signal	Size
DEI-Campaign A	19–22 March 2009	Temperature	29 nodes × 781
OrangeLab-Campaign A	26–27 August 2008	Temperature	75 nodes × 65
EPFL-Campaign A	12–15 January 2007	Soil moisture	20 nodes × 742
DEI-Campaign B	19–22 March 2009	Voltage	45 nodes × 754

**Table 3:** Performance evaluations of different sensory datasets.

Sparse Basis	GI	NS
(a) Temperature of DEI-Campaign A		
spatial	0.0118	780.6154
DCT	0.2526	13.3167
haar-1	0.5077	391.4284
haar-2	0.7566	196.1361
rbio5.5	0.5268	395.8623
OBA	0.7842	2.8856
(b) Temperature of OrangeLab-Campaign A		
spatial	0.0292	64.8315
DCT	0.3313	6.8109
haar-1	0.4895	33.3158
haar-2	0.7585	18.5259
rbio5.5	0.6940	39.4684
OBA	0.7809	1.7180
(c) Soil moisture of EPFL-Campaign A		
spatial	0.4942	741.6826
DCT	0.2266	13.3031
haar-1	0.5022	373.4321
haar-2	0.7478	188.5487
rbio5.5	0.5227	378.0363
OBA	0.7496	2.9541
(d) Voltage of DEI-Campaign B		
spatial	2.5089 × 10^−4^	753.9996
DCT	0.2293	13.6672
haar-1	0.5000	377.1115
haar-2	0.7516	189.1442
rbio5.5	0.5196	382.1088
OBA	0.9820	1.0324

**Table 4:** Reconstruction errors of four different datasets vs. measurement M for BPDN.

Temperature of DEI-Campaign A (d′=60, K=60, Flen=781)									
M	200	250	300	350	400	450	500	550	600
error	1.2909	1.0517	0.9592	0.7463	0.7262	0.6792	0.5919	0.5224	0.4385
Temperature of OrangeLab-Campaign A (d′=10, K=30, Flen=64)									
M	10	15	20	25	30	35	40	45	50
error	2.7764	1.2690	1.0621	0.9650	0.8080	0.7198	0.6355	0.5000	0.4731
Soil moisture of EPFL-Campaign A (d′=60, K=60, Flen=128)									
M	20	30	40	50	60	70	80	90	100
error	1.5136	1.4068	1.1020	1.0268	0.9443	0.7936	0.6169	0.5336	0.4154
Voltage of DEI-Campaign B (d′=60, K=60, Flen=128)									
M	20	30	40	50	60	70	80	90	100
error	1.5541	1.3264	1.2549	0.9252	0.8494	0.7387	0.5565	0.5427	0.3943

**Table 5:** Reconstruction errors of four different datasets vs. measurement M for GOMP.

Temperature of DEI-Campaign A (d′=60, K=60, Flen=781)									
M	200	250	300	350	400	450	500	550	600
error	1.9917	1.8461	1.6777	1.5731	1.4157	1.3460	1.0983	0.9937	0.9475
Temperature of OrangeLab-Campaign A (d′=10, K=30, Flen=64)									
M	10	15	20	25	30	35	40	45	50
error	10.4145	1.7442	1.2315	1.1896	0.9481	0.8384	0.9338	0.7394	0.6125
Soil moisture of EPFL-Campaign A (d′=60, K=60, Flen=128)									
M	20	30	40	50	60	70	80	90	100
error	1.7484	1.7685	1.3382	1.2300	1.3735	1.0918	0.9362	0.8433	0.7216
Voltage of DEI-Campaign B (d′=60, K=60, , Flen=128)									
M	20	30	40	50	60	70	80	90	100
error	1.6838	1.4522	1.4358	1.2890	1.1972	1.0570	0.9642	0.7258	0.7119

## Data Availability

EPFL LUCE SensorScope WSN: http://sensorscope. epfl.ch/.
